# 

**Published:** 1886-10

**Authors:** 


| | Page |
|---|---|
| **Original Communications:** | |
| Pharyngeal and Nasal Surgery by the Galvano-Cautery. W. E. Casselberry | 333 |
| Favus. H. J. Reynolds | 344 |
| A Case of Actinomycosis Hominis. A. Schirmer | 354 |
| **Editorial:** | |
| Asepsis versus Antisepsis | 356 |
| Actinomycosis | 358 |
| **Foreign Correspondence:** | |
| Clinical Notes from Vienna. W. S. Caldwell | 360 |
| **Society Reports:** | |
| *Chicago Medical Society; Meeting August 16.* Membranous Cast of the Trachea and Larynx. F. E. Waxham | 370 |
| Radical Cure of Inguinal Hernia. J. B. Hamilton | 371 |
| Supra-Pubic Cystotomy. W. T. Belfield | 379 |
| Obituary. Professor Frank H. Hamilton | 381 |
| *Meeting September 6.* Overfilling and Dilatation of the Colon. J. S. Jewell | 381 |
| Actinomycosis Hominis. A. Schirmer | 389 |
| Caries of the Right Parietal Bone. A. V. Park | 390 |
| *Chicago Gynæcological Society; Meeting July 16.* Occlusion of the Os Uteri as an Impediment to Labour. F. E. Waxham | 391 |
| Cause and Treatment of Pelvic Hæmatocele | 396 |
| **Abstract:** | |
| St. Bartholomew's Hospital Reports. | 405 |
| Radical Cure of Oblique Inguinal Hernia by Internal Abdominal Peritoneal Pad. W. MacEwen | 411 |
| **Book Reviews:** | |
| Diseases of the Digestive Organs in Infancy and Childhood. L. Starr. | 414 |
| Elements of Surgical Diagnosis. A. P. Gould | 417 |
| *Manuel des Injections Sous-Cutanées* | 417 |
| *Manuel de Technique des Autopsies.* P. Bricon | 418 |
| The Blot Upon the Brain. W. W. Ireland | 419 |
| *Traite Elementaire d'Anatomie Medical du Systeme Nerveux.* C. Fere | 419 |
| A Manual of Therapeutics. E. J. Waring | 420 |
| Medical and Surgical Directory of the United States. R. L. Polk | 420 |
| The Methods of Bacteriological Investigation. F. Hueppe | 421 |
| Essentials of Vaccination. W. A. Hardaway | 422 |
| The Best Preliminary Education for the Study of Medicine. E. S. Stevens | 423 |
| Practical Human Anatomy. F. D. Weisse | 424 |
| A Manual of Ausculation and Percussion. A. Flint | 425 |
| Manual of Operative Surgery. L. A. Stimson | 426 |
| Books Received | 427 |
| Pamphlets Received | 428 |

